# Chameleon sequences reveal structural effects in proteins representing micelle‐like distribution of hydrophobicity

**DOI:** 10.1002/2211-5463.70214

**Published:** 2026-02-17

**Authors:** Irena Roterman, Katarzyna Stapor, Leszek Konieczny, Krzysztof Gądek, Piotr Nowakowski

**Affiliations:** ^1^ Department of Bioinformatics and Telemedicine Jagiellonian University – Medical College Krakow Poland; ^2^ Faculty of Automatic, Electronics and Computer Science, Department of Applied Informatics Silesian University of Technology Gliwice Poland; ^3^ Chair of Medical Biochemistry Jagiellonian University – Medical College Krakow Poland; ^4^ Sano Centre for Computational Medicine Kraków Poland

**Keywords:** chameleon proteins, hydrophobicity, local environment, protein folding, secondary structure, structure prediction

## Abstract

The fundamental principle for protein structuring is that the 3D structure is determined by the amino acid sequence. An important phenomenon in this regard is the presence of a differentiated secondary structure for chain fragments of identical sequence—referred to as chameleons. In the present work, individual chain fragments were shown to constitute components of a system with specific physicochemical properties of the overall structure, adapting the secondary structure to the superior role assigned to the protein. The proteins (domains) represent the construction with a centrally located hydrophobic core and a polar surface (described by 3D Gauss function) representing a favourable arrangement in contact with water—the standard environment for the activity of most proteins. The fuzzy oil drop model (FOD‐M), which takes into account the presence of a non‐water environment for the active protein, was used to assess this condition. The proteins present in the ChSeq database are the object of analysis. The criterion for qualifying a pair of proteins for the analysis presented herein is the presence of a structuring—a hydrophobicity distribution consistent with a 3D Gaussian distribution representing a micelle‐like system. The analysis shows that an appropriate secondary structure is not an aim by itself, but a means to achieve a hydrophobicity distribution appropriate for the status represented by the structural unit with its defined biological activity. The mathematical model for *in silico* simulation of protein folding process as environment dependent is also presented.

## Graphical abstract

Amino acids sequence of two different proteins with the same sequence (chameleon sequence—black boxes) represent in 3D structure of the proteins different secondary structures: HHHH—helical and BBB—Beta‐structural. The chains folded in water environment adopt different III‐order structures in which the chameleon fragments appear to adopt similar status in respect to hydrophobicity distribution in the protein body.

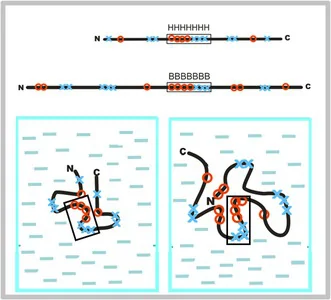

**Abbreviations**
3D: spatial; three dimensional
3D Gauss: 3‐dimensional form of Gauss function
*D*_KL_
: Kullback–Leibler divergence entropy
FOD‐M: fuzzy oil drop model modified
*K*
: parameter modifying the 3D Gauss function
*M*
: modified distribution of theoretical hydrophobicity
*O*
: distribution of observed hydrophobicity calculated as interresidual hydrophobic interaction
*O*/*R*
: relations between target distribution of hydrophobicity (*O*) in respect to *R* distribution—unified hydrophobicity distribution
*O*/*T*
: relations of observed (target distribution) versus theoretical (*T*) hydrophobicity distribution
PDB: Protein Data Bank
PDB ID: Protein Data Bank Identification
*R*
: unified hydrophobicity distribution
RD: relative distance
*T*
: theoretical distribution of hydrophobicity accordant with 3D Gauss function spanned all over the protein molecule

Proteins containing chameleon sequences are the subject of numerous analyses [1, 2]. The technique of infinite‐swap replica exchange incorporated into molecular dynamics simulation appears to be the convenient tool for fast structural sampling in changing conditions (temperature) to reveal the preferences for structure stabilisation [3, 4]. Modifications in procedures oriented on protein folding simulation are focused on conformational space in search for energetically optimal organisation in respect to secondary structure determination [5, 6]. A unique arrangement for Beta‐hairpin generation has been defined as initiating ordering already in the early stages of folding [7]. The role of the loop structure is proving critical to the folding process [8]. The importance of chameleon sections in amyloid‐transformed protein chains is discussed in [9].

The characteristics of proteins (structural units: chains or domains) are focused in this paper on the hydrophobicity distribution in protein body [10, 11]. The participation of chameleon fragments in the construction of the complete structural units (chameleon protein pairs) is assessed to verify the hypothesis of superior role of the whole body construction in respect to the secondary role of fragments fitting their secondary structure to the main goal which is the structure ensuring the biological activity. Analysis of evolution‐based structural changes limited to secondary and domain construction is presented in [12]. No evolution‐related status of chameleon proteins is available so far.

Analysis presented in this paper is focused on the active role of environment directing the protein folding process towards the form appropriate for specific local surrounding. The differences of these roles can be easily recognised comparing the water‐soluble proteins versus the membrane proteins. Water environment directs the folding process towards the hydrophobic core formation with polar exposure on surface. The membrane environment directs the folding process toward the exposure of hydrophobic residues on the surface to ensure the stability in hydrophobic surrounding in the membrane. The trans‐membrane protein playing the role of channels additionally concentrates the polar residues in central part of the protein body. These two examples visualise the opposite role of environment in protein folding. The differences can be identified using the FOD‐M model examining the hydrophobicity distribution in quantitative form. This is why the definition of the status of chameleon sequences adopting different secondary structure by the fragments of same amino acid sequence is aimed at the examination of their role in hydrophobicity distribution. It is shown in this paper that the hydrophobicity distribution is the prior factor for the folding protein in respect to selection of secondary structure. The secondary structure is just the structural form to reach appropriate status in respect to hydrophobicity distribution in protein body.

## Materials and methods

### Description of the FOD‐M model used

The FOD‐M model [10, 11] was used to analyse the status of the structural units in respect to hydrophobicity distribution. The basis for this model is the assumption that the bi‐polar molecules—as all amino acids are with different relations between polar and non‐polar—tend to construct the forms of micelle‐like ordering of hydrophobicity. Micelle form means high concentration of hydrophobicity in the central part with a polar surface ensuring solubility in a water environment. The 3D Gauss function is able to describe this type of hydrophobicity distribution. It is assumed that the folding protein fits its structure to the ordering preferable for a water environment. Due to the significant mobility limitation of amino acids in the polypeptide chain by the covalent bonds, the chain is able to re‐construct the micelle organisation only to a limited degree. However, the reference distribution expressed by the 3D Gauss function can be applied to any proteins in the procedure of encapsulating the protein body in this function. The 3D Gauss function represents the idealised distribution and is treated as the reference distribution. The real, observed hydrophobicity distribution as appears in a certain protein compared with the reference distribution can measure the degree of similarity. The comparable analysis allows identification of residues representing the local disorder in respect to micelle‐like organisation. The residues following the micelle‐like distribution give the information about the part of the protein responsible for solubility and hydrophobic core formation. On the other hand, the residues revealing the status of local disorder appear to represent the coded form expressing very frequently the specificity of the protein under consideration [10, 11]. Local deficiency of hydrophobicity indicates the presence of a cavity. Local excess of hydrophobicity—especially when it occurs on the surface—can be interpreted as an area ready to interact with other proteins of similar characteristics.

The hydrophobicity in a protein molecule can be assessed by two functions:

1. The observed distribution—*Oi*—Resulting from hydrophobic inter‐amino acid interactions. The function introduced by M. Levitt [13] is used in this case, where the interaction is expressed by the function:

(1) $$H_{i}^{O}=\frac{1}{H_{\mathrm{sum}}^{O}}\sum_{j}\{\begin{array}{l} \left(H_{i}^{r}+H_{j}^{r}\right)\left(1-\frac{1}{2}\left(7{\left(\frac{r_{\mathrm{ij}}}{c}\right)}^{2}-9{\left(\frac{r_{\mathrm{ij}}}{c}\right)}^{4}+5{\left(\frac{r_{\mathrm{ij}}}{c}\right)}^{6}-{\left(\frac{r_{\mathrm{ij}}}{c}\right)}^{8}\right)\right)\;\text{for}\;r_{\mathrm{ij}}\leq c\\ 0,\;\text{for}\;r_{\mathrm{ij}}>c \end{array}$$

The positions of individual amino acids are represented by the positions of the so‐called effective atoms (the averaged position of the atoms comprising a given amino acid). As can be seen from the equation, the magnitude of such an interaction depends on the distance between the amino acids *r*_*ij*_ and on their intrinsic hydrophobicity (*H* ^*r*^)—any scale can be applied. The application of Kyte‐Doolittle was applied in calculations presented in to make the comparison of these two scales [14]. The *c* parameter expresses the cutoff distance for hydrophobic interaction – according to [13] *c* = 9 Å. The form of this function is the empirical one as proposed in [13].

2 The theoretical distribution—*T*_*i*_—expresses an idealised distribution fulfilling the criteria of a micelle‐like system with a centrally located core and a gradually decreasing level of hydrophobicity up to zero at the surface. This distribution is expressed by a 3D Gaussian function spanning the protein body. The value of this function at the position of the effective atom expresses the expected idealised level of hydrophobicity—called *T*_*i*_– at a given location of the protein.

(2) $$\begin{array}{rcl} T_{i} & = & H_{i}^{T}\\ & = & \frac{1}{H_{\mathrm{sum}}^{T}}\exp\left(\frac{-{\left(x_{i}-\bar{x}\right)}^{2}}{2\sigma_{x}^{2}}\right)\exp\left(\frac{-{\left(y_{i}-\bar{y}\right)}^{2}}{2\sigma_{y}^{2}}\right)\exp\left(\frac{-{\left(z_{i}-\bar{z}\right)}^{2}}{2\sigma_{z}^{2}}\right) \end{array}$$

The values of the parameters *σ*_*x*_, *σ*_*y*_ and *σ*_*z*_ are adjusted to the size and shape of the protein examined. The visualisation of encapsulation is given in Fig. 1E

**Fig. 1:**
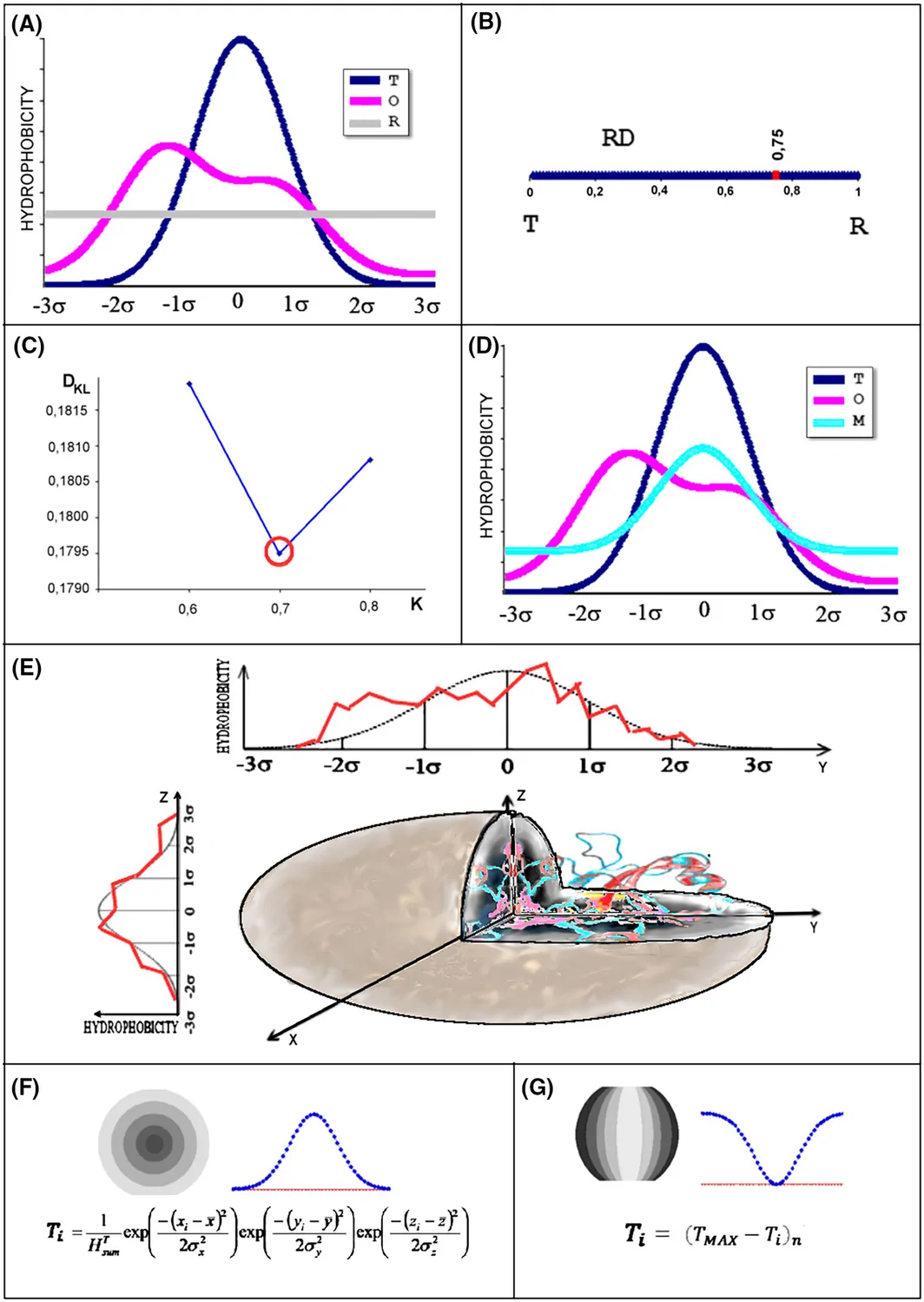
Visualisation of the model used. (A) example of *T* (blue), *O* (pink) and *R* (grey) distribution—form reduced to 1D. (B) determined value of the parameter RD = 0.75 suggesting the closeness of the *O* distribution to the *R* distribution. The presence of a hydrophobic core in the hypothetical *O* distribution is not identified. (C) method of determining the value of the parameter *K* = 0.7 – value corresponds to the minimum *D*_KL_ for the (*O|M*) relationship. (D) *T* (blue), *O* (pink) and *M* (light blue) distributions for the value of *K* = 0.7 determined as in C. (E) protein encapsulated in 3D Gauss function (black line) with observed distribution (red line) – hypothetical distribution. The 3D structure generated using the VMD program [31, 49]. (F) mathematical expression of polar environment – water (Gauss function) influencing protein folding towards the generation of hydrophobic core and polar surface. The more dark colour, the higher hydrophobicity. (G) mathematical expression for membrane environment with hydrophobic residues exposed on the surface with polar central part as it is observed in trans‐membrane channels.

Normalisation of both distributions (Σ*H* ^o^ = 1.0 and Σ*H* ^*T*^ = 1.0) allows the quantitative assessment of similarity between these two profiles.

This assessment is performed using the divergence entropy introduced by Kullback–Leibler [15].

(3) $$D_{\mathrm{KL}}\left(P|Q\right)=\sum_{i=1}^{N}P_{i}\log_{2}\frac{P_{i}}{Q_{i}}$$

Where the *P* distribution—the distribution examined—in the case of the FOD model this is the *O* distribution, the *Q* distribution is the reference distribution, in the FOD model—the *T* distribution.

The determined *D*_KL_ value for the relationship (*O|T*) is not interpretable. Therefore, a second reference distribution *R* is introduced with *R*_*i*_ = 1/*N*, where *N* is the number of amino acids in the chain.

The *D*_KL_ value for the (*O|R*) relationship determines the degree of closeness of the *O* distribution to a distribution devoid of the presence of a hydrophobic core (uniform distribution throughout the protein). The visualisation of *T*, *O* and *R* distribution is given in Fig. 1A

The relationship *D*_KL_(*O|T*) < *D*_KL_(*O|R*) indicates the presence of a hydrophobic core. The degree of fit of the *O* distribution to the *T* distribution is expressed by the RD (Relative Distance) parameter determined according to the equation:

(4) $$\mathrm{RD}=\frac{D_{\mathrm{KL}}\left(O|T\right)}{D_{\mathrm{KL}}\left(O|T\right)+D_{\mathrm{KL}}\left(O|R\right)}.$$

A value of RD < 0.5 is interpreted as the presence of a hydrophobic core.

In the analysis so far, proteins with a low RD value have been identified, which provides evidence for the correctness of the proposed model (Fig. 1B). These proteins are: downhill, fast‐folding, ultra‐fast‐folding and antifreeze type II proteins [16].

It should be noted that the model in question can be applied to any defined structural unit: a multi‐chain complex, a single chain or a domain. A 3D Gaussian function is generated for each listed structural unit. It is also possible to determine the status of a selected chain section and its importance (role) in the construction of a micelle‐like distribution within a given structural unit. A value of RD < 0.5 for the selected fragment (this can be the status of a single chain in a complex or a chain fragment against a randomly selected structural unit) indicates the participation of this fragment in the construction of a generally unit hydrophobic core. A value of RD > 0.5 for a selected chain fragment indicates its local inadequacy in the construction of the hydrophobic core common for the entire unit. When analysing the status of a selected structural unit fragment, the set of *O*_*i*_, *T*_*i*_ and *R*_*i*_ values is subjected to a normalisation procedure.

The model discussed herein has been used to assess the status of a structural unit where a fragment identified as chameleon is present and the status of the section itself with identical sequence in two different proteins. The structural units used in the present analysis follow the CATH classification [17].

The importance of micelle‐like ordering of hydrophobicity is strongly related to the water environment which is the basic condition for all biological systems in Nature. This is why the analysis of water participation in the protein structuralisation (folding) is of high importance. The identification of any form of hydrophobicity distribution similarity in respect to the water‐preferable one is the object of analysis. In this context, the status of chameleon fragments is analysed especially.

The FOD‐M model takes into account the presence of a non‐aqueous environment for a given protein. The degree of mismatch of the *O* distribution against the *T* distribution may be due to the different characteristics of the environment, which, by actively participating in protein folding, may lead to a status different from that obtained in the presence of water. The construction of a centric core with a polar shell is characteristic of an aqueous environment interacting with bi‐polar molecules, which is what all amino acids are (with a variable ratio of polar to hydrophobic parts).

The presence and influence of the external field on the formation of the hydrophobicity distribution within the protein molecule was defined starting from the environment of the membrane, which, as opposed to the water environment, expects the exposure of hydrophobic amino acids on the surface (contact with the amphipathic membrane) and (especially in the case of membrane proteins responsible for transport) the concentration of polar residues in the central part of the protein (Fig. 1F). Therefore, the description of the force field in a membrane protein is expressed by a function complementary to the Gaussian function:

(5) $$\mathrm{Mi}=T_{\max}-\mathrm{Ti}$$

In fact, the analysis of membrane proteins suggests the use of the following equation to record the force field based on hydrophobicity:

(6) $$\mathrm{Mi}=\mathrm{Ti}+\left[K*\left(T_{\max}-T_{i}\right)n\right]n$$

In this field record, there is a water‐derived field (3D Gauss function) modified by the presence of a complement field (*T*_max_ – *T*_*i*_) to a degree expressed by the value of the parameter *K*. The parameter *K* can take the value *K* = 0.0 for proteins with a hydrophobicity structure compatible with a centric core and a polar surface up to a value *K* > 3 in some membrane proteins. The minimal *D*_KL_(*O|M*) for different *K* values identifies the proper *K* value for the case under consideration. The graphic visualisation of the model is shown in Fig. 1. The *K* values can be found in iteration procedure searching for *K* value for which the *D*_KL_(*O*|*M*) reaches minimum (Fig. 1C). The minimum is assumed to represent the form of *M* function (the modified *T* function) to which the folding protein tries to fit during the folding process. The parameter *K* expresses the participation of other compound (not solely water) changing the characteristics of environment and thus influencing the folding process (Fig. 1D).

The interpretation of the FOD‐M model parameters is as follows:

1. The RD value for the structural unit indicates the extent to which the micelle‐like system has been reproduced in the protein structure. High RD values indicate an approximation to a uniform distribution, devoid of centralisation of hydrophobicity in the protein.
  - The *T* and *O* distributions are determined to obtain the RD value.
2. The value of *K* determines the degree of contribution of non‐aqueous environmental factors changing the specificity of the aqueous environment by introducing a hydrophobic factor in particular. The *M* profile is determined expressing the variability of the theoretical distribution closest to the *O* distribution.
3. The RD value for the chameleon section determines its local status within a structural unit based on the relationship of the *T* and *O* distributions. A high RD value expresses a local mismatch to the micelle‐like system.

All proteins analysed here are characterised according to the following pattern:

1. A structural unit is identified within which a chameleon fragment is present (according to the CATH classification [17]) available in the PDBSum database [18].
2. For the identified structural unit (chain or domain), the RD value is determined to obtain the degree of micelle‐like system reconstruction.
3. For each unit, the *K* value is determined to identify the possible influence of the environment on the formation of the structure of the unit in question.
4. For a chameleon section, the RD value is determined to identify the contribution of the fragment in the structure of the hydrophobic core within the structural unit.

The RD values for pairs of chameleon proteins are presented in the form of scatter diagrams expressing the relationship of the status of both structural units in chameleon proteins pairs and scatter diagrams for chameleon sections representing distinct forms of secondary structure. A correlation coefficient is determined to express a measure of the consistency of the status of a given section with the whole structure within a given structural unit.

The visualisation of the influence of the environment on the folding process given in Fig. 2 reveals the orientation of structuring depending on the specific environment, which is water as discussed in this paper. It applies to the aqueous environment directing the structuring generating the presence of a hydrophobic core. The location and corresponding adjustment of the chameleon sections to the structuring unit imply a similar status in both proteins compared regardless of the sequence specificity and chain length. The groups of other sets of chameleon proteins are discussed in [19, 20].

**Fig. 2:**
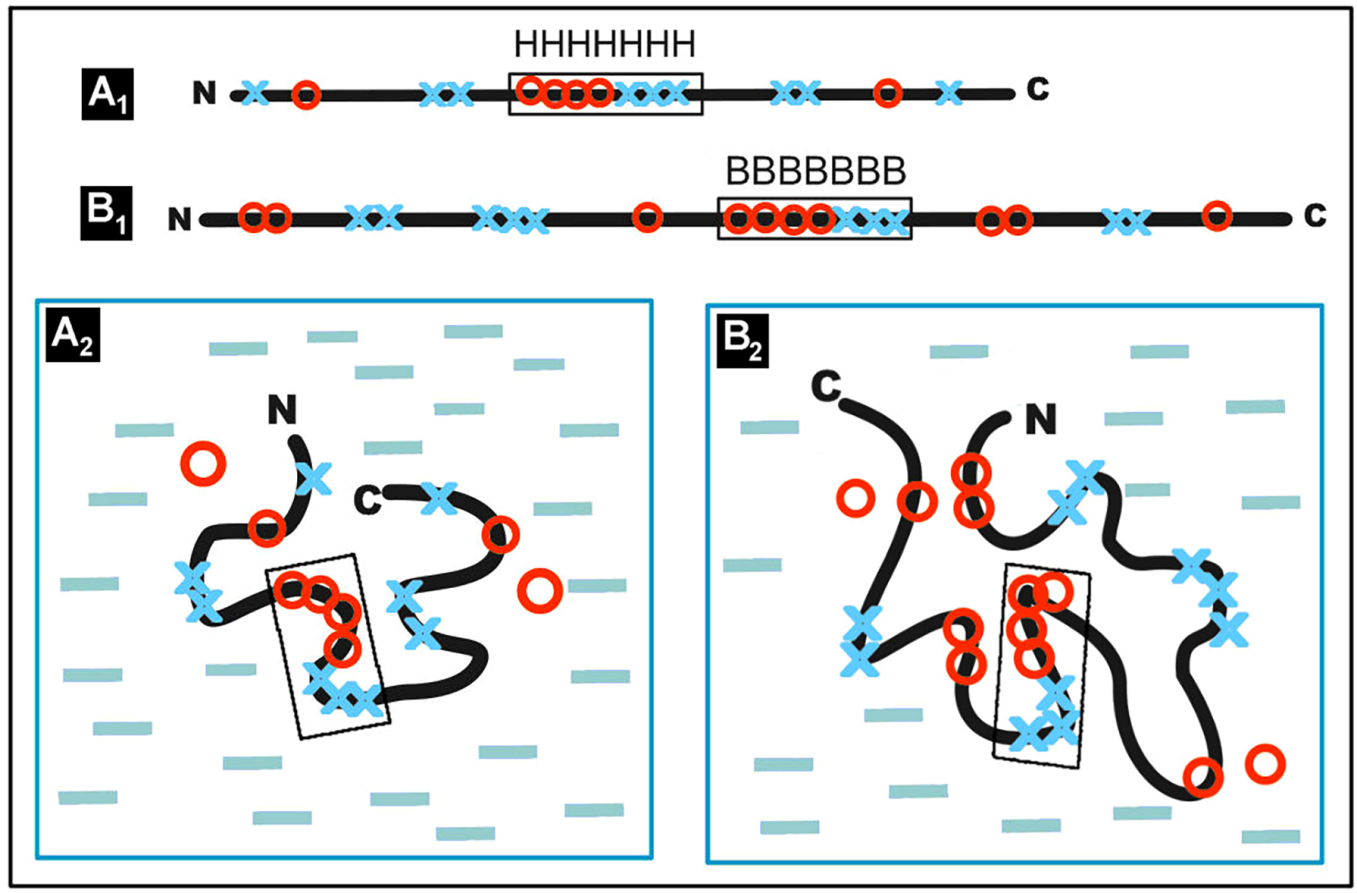
Summary of the influence of environmental specificity on the structuring of a polypeptide chain with a hydrophobicity (red O)/polarity (blue X) distribution of amino acids. The black frames distinguish the chameleon fragments.

The description of the model may appear similar to the presentation given in [20] due to the applicability of the same model; however, the object of analysis is different.

Visualisation given in Fig. 2. is limited to proteins with hydrophobicity distribution similar to micelle‐like (red circles representing hydrophobic residues concentrated in central part and hydrophilic ones—blue X—exposed on the surface) representing the set of proteins selected for analysis in this paper.

The program to calculate the RD and *K* parameters is freely available https://hphob.sano.science.

### Data

The ChSeq database available at prodata.swmed.edu/chseq was used as the source for pairs of proteins with chameleon sequences present representing different: helical and β‐structural forms for chameleon sequence fragments. The structure was obtained from the PDB database [21].

The sub‐base for non‐homologous proteins with 8 aa (24 pairs of proteins) and 7 aa (777 pairs of proteins) was generated classified by a stringent criterion (ChSeq database – [22, 23]). The sub‐base was extracted from the aforementioned ChSeq database according to a criterion expressed as RD < 0.5 for both proteins in the pair. The analysed sub‐base is composed of 116 pairs of proteins.

The aim of the analysis is to assess the status of chameleon fragments in respect to the structure of the structural unit. The hypothesis is assumed that the secondary structures adopted by certain chain fragments depend on the whole structural unit organisation. The secondary structure is not an aim by itself. The scatter diagram representing the status (expressed by RD) of chameleon fragments is to check the accordance for two fragments of different secondary structure. Some outstanding points in the scatter presentation can be distinguished. This unusual status of these examples is discussed in detail. The list of these proteins is given in Table 1.

**Table 1:** The list of proteins present in the detailed analysis in this paper with a short characterisation. Selection of proteins explained on Fig. 3C.

Beta‐structure			Sequence	Helix		
PDB ID	Function	Source organism		Source organism	Function	PDB ID
1‐1I7O [50]	Isomerase/lyase	*E. coli*	IAGYALA	Human	Immune	2A73‐B [51]
2‐1Z0A [52]	Transport	Ras‐related protein rab‐2a	AAGALLV	Plant	Hydrolase	3N1D [53]
3‐3BBN‐K [54]	Ribosome	Plant‐spinach	LLSFVRD	Human	Metal transport	1CX8 [55]
4‐1T3E [56]	Glycine receptor	Rabbit	GKIRDSNRS	Protozoa	Membrane prot	1S0P‐A [57]
5‐4J56‐E [58]	Oxidoreductase	Parasite	SSVDTLL	Plant	Unknown	1WIV [59]
6‐3N05 [60]	Ligase	Bacteria	LVGYLDR	Human	Lipid bonding	2RB3 [61]

## Results

In the entire set of proteins analysed (ChSeq database), 116 part proteins fulfilling the RD < 0.5 condition in both proteins constituting a pair representing the helical structure and β‐structure for the chameleon section were identified.

Questions addressed to the analysed proteins:

1. to what extent do chameleon sections with a different secondary structure fit into the overall structure of a given structural unit?
2. is the local status of chameleon sections comparable to the status of the structural unit (protein/domain)?
3. to what extent is the status of chameleon sections variable compared to the status of the structural unit as a whole?

The answers to the questions posed will be formulated in the fuzzy oil drop model convention and thus expressed in terms of the RD and *K* parameter values derived from the characteristics of the *T* and *O* as well as *M* profiles.

It should be noted that the selected group of proteins is those representing a micelle‐like system with a centrally located hydrophobic core and a polar shell.

The status of the structural units was restricted to pairs of proteins meeting the RD < 0.5 condition in both cases (Fig. 3A – red dots). The status of the chameleon sections demonstrates a significant scatter (Fig. 3A – blue dots). The value of the correlation coefficient for the relationship between the status of the chameleon sections is 0. 217. The analysis of the status relationship (expressed by FOD‐based parameters) can be performed based on an ordered set, where the higher status RD values are placed on the horizontal axis, while the status of the chameleon section with the lower value in the pair of chameleon proteins is placed on the vertical axis (Fig. 3B). The scatter of dots in this arrangement will be expressed by the value of correlation coefficient = 0.589 (all dots representing the status of chameleon sections). The subjective elimination of 18 outlier dots, accounting for 15% of the total analysed set, increased the value of the correlation coefficient to 0.840 (Fig. 3B – blue dots—eliminated). This operation aims to identify proteins that significantly show a different status of chameleon sections depending on their secondary structure.

**Fig. 3:**
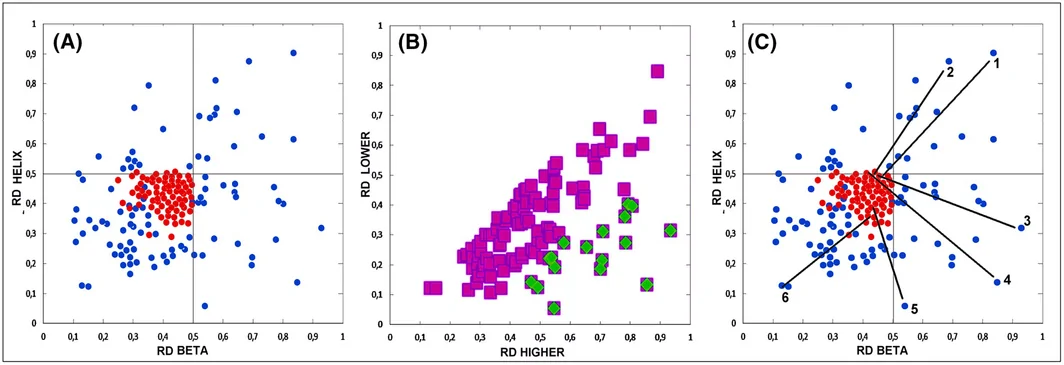
Characteristics of the analysed set of chameleon proteins: (A) status of structural units (red dots) containing chameleon sections—blue dots in the system: *X*‐axis—β‐structure, *Y* axis—helix. (B) ordered arrangement—horizontal axis—RD values higher for the chameleon section from the given pair, vertical axis—RD values lower in the given pair—here the ordering of the helix versus β‐structure relationship is not present. Colour variation: green dots—dots treated as outliers. The magenta points represent 85% of the set analysed in this paper. The correlation coefficient for magenta point is equal to 0.85. (C) selection of extreme examples representing the differential status of chameleon sections – subsequent numbers represent pairs discussed in detail later in the paper. The analysis is designed to identify the varied status of the chameleon sections. The red points—status of chameleon pairs of domains. It visualises the selection of the sub‐group from ChSeq—both domains represent the status expressed by RD < 0.5 as also shown in A.

A collective summary of the selected protein pairs is presented in Table 2.

**Table 2:** Summary of results characterising the status of selected examples (Fig. 3C). The left column shows the identifier consistent with the numbering introduced in Fig. 3C.

Beta‐structure				Sequence	Helix			
PDB ID	Function	RD/*K*			RD/*K*		Function	PDB ID
		Domain	Fr		Fr	Domain		
1‐1I7O	Isomerase/lyase	0.458/0.32	0.848	IAGYALA	0.893	0.467/0.34	Immune	2A73‐B
2‐1Z0A	Transport	0.440/0.33	0.696	AAGALLV	0.868	0.480/0.34	Hydrolase	3N1D
3‐3BBN	Ribosome	0.410/0.24	0.938	LLSFVRD	0.314	0.460/0.28	Metal transport	1CX8
4‐1T3E	Glycine recept	0.400/0.23	0.857	GKIRDSNRS	0.133	0.470/0.40	Membrane prot	1S0P‐A
5‐4J56E	Oxidoreductase	0.450/0.27	0.558	SSVDTLL	0.053	0.380/0.10	Unknown	1WIV
6‐3N05	Ligase	0.438/0.33	0.157	LVGYLDR	0.120	0.330/0.05	Lipid bonding	2RB3

### Examples of proteins showing highly similar status of chameleon sections with extremely high RD values in both proteins

Two pairs of chameleon proteins are distinguished by a very high consistency of chameleon section status at a very high RD value (points 1 and 2 on Fig. 3C). These two examples demonstrate that micelle‐like structural units (central hydrophobic core with polar surface) in their structure contain sections with a local mismatch towards such an arrangement (a high RD value for chameleon sections). Characteristically, however, the statuses of chameleon sections in pairs are highly comparable in both cases. This is illustrated by the sets of the *T* and *O* profiles for chameleon sections (Figs 4E,F, 5E,F). It should be noted that this status is comparable regardless of the biological activity of the protein pairs and of the varied chain length (as in the case of 1I7O and 2A73; Fig. 4A–D).

**Fig. 4:**
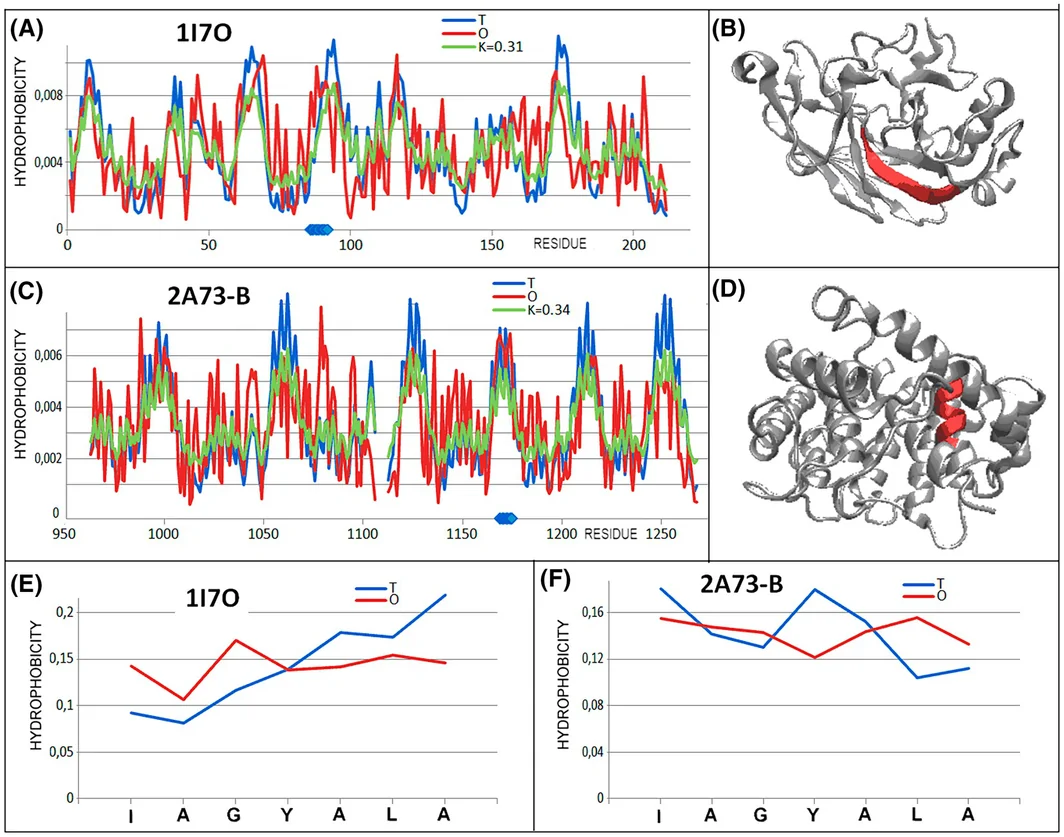
Pair characterisation (Position 1 in Fig. 3C position 227 on the ChSeq database). (A) distribution for the *K* value (given in the legend) describing the isomerase/lyase status (1I7O). (B) 3D representation of the structural unit for which the parameters have been determined with the chameleon section (red) highlighted – 1I7O. (C) *T* (blue), *O* (red), and *M* (green) distribution for the *K* value (given in the legend) describing the status of protein belonging to the immune system (2A73‐B). (D) 3D representation of the structural unit for which the parameters have been determined with the chameleon section (red) highlighted – 2A73‐B. (E) *T* (blue) and *O* (red) distribution for the chameleon section in isomerase/lyase (1I7O). (F) *T* (blue) and *O* (red) distributions for the chameleon section in hydrolase (2A73‐B). The blue dots on the *X*‐axis in A and C—chameleon fragment. The 3D structures generated using the VMD program [31, 49].

**Fig. 5:**
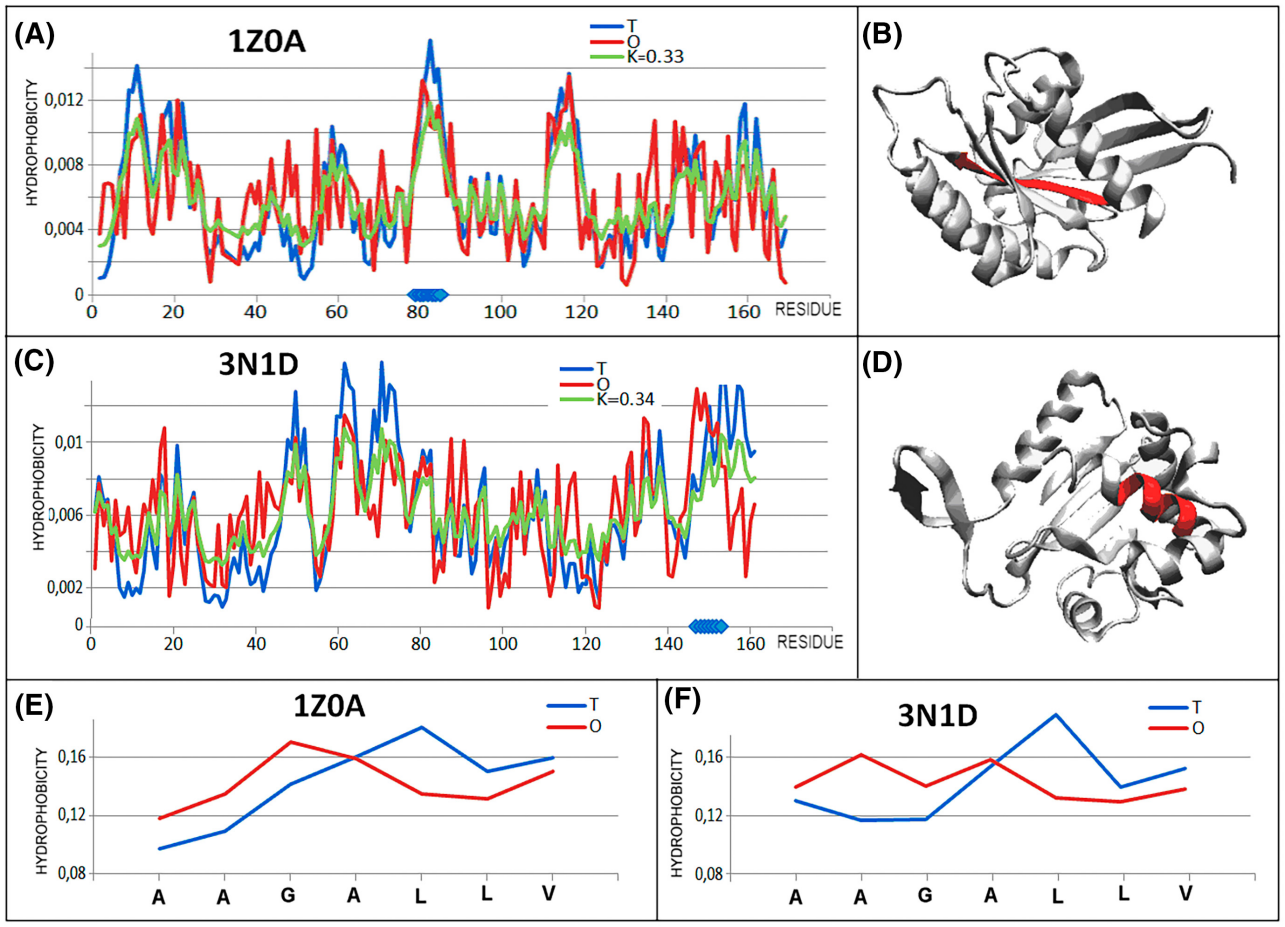
Pair characterisation (Position 2 in Fig. 3C position 681 on the ChSeq database). (A) *T* (blue), *O* (red), and *M* (green) distributions for *K* value (given in the legend) describing the status of protein engaged in transport (1Z0A). (B) 3D representation of the structural unit for which the parameters have been determined with the chameleon section (red) highlighted – 1Z0A. (C) *T* (blue), *O* (red), and *M* (green) distribution for the *K* value (given in the legend) describing the status of hydrolase (3N1D). (D) 3D representation of the structural unit for which the parameters have been determined with the chameleon section (red) highlighted—3N1D. (E) *T* (blue) and *O* (red) distribution for the chameleon section in 1Z0A. (F) *T* (blue) and *O* (red) distributions for the chameleon section in hydrolase (3N1D). The blue dots on the *X*‐axis in A and C—chameleon fragment. The 3D structures generated using the VMD program [31, 49].

### Examples of pairs of proteins with different chameleon section status

As shown in Fig. 3C the pairs: 3 – 3BBN‐K—ribosome component and 1CX8—metal transport protein and 4 – 4J56‐E – oxidoreductase and 1WIV – protein of unknown activity, the status of chameleon sections is significantly different. The structural units represent, in these two cases, a very high ordering consistent with a micelle‐like arrangement (*K* values < 0.3), containing in their structure chameleon sections of significantly different status. This is revealed by the sets of profiles in Figs 6A,C, 7A,C. The variation in the status of the chameleon sections illustrated in Figs 6E,F, 7E,F reveals a high mismatch between the *O* distribution and the *T* distribution for 3BBN‐K. The protein, which is a component of the ribosome, interacts with numerous different molecules affecting the local type of ordering.

**Fig. 6:**
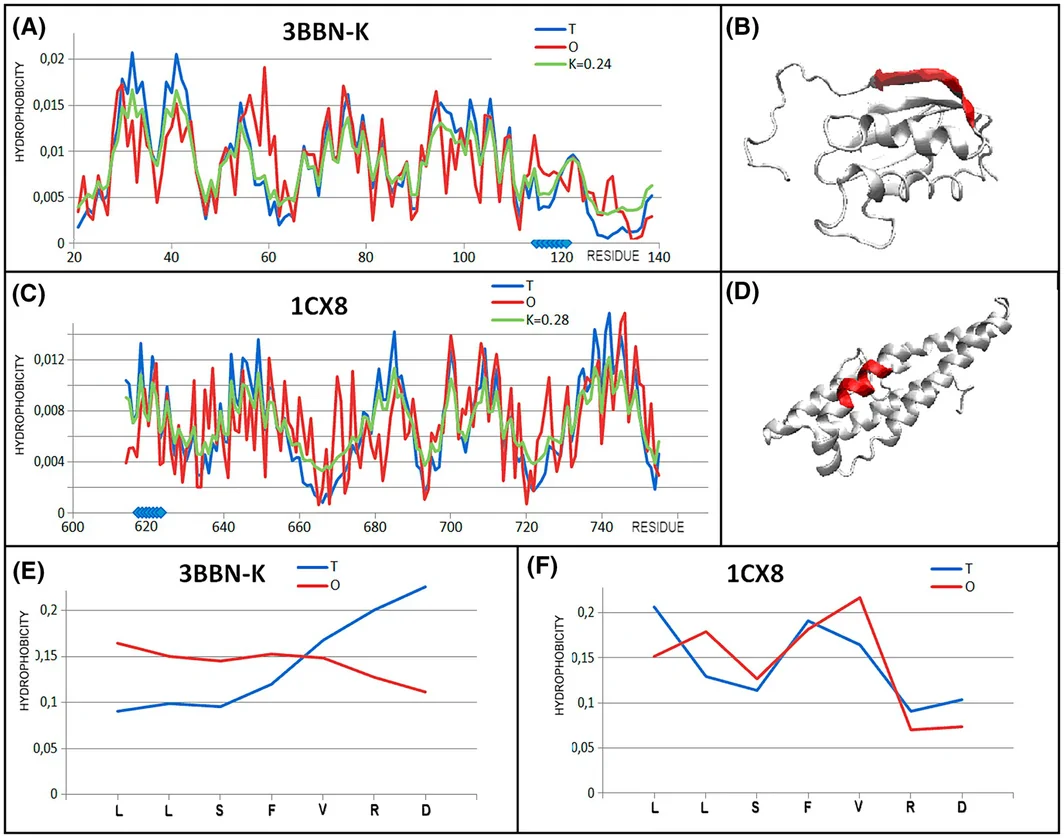
Pair characterisation (position 3 in Fig. 3C position 764 on the ChSeq database). (A) *T* (blue), *O* (red), and *M* (green) distribution for the *K* value (given in the legend) describing the status of protein – component of ribosome (3BBN‐K). (B) 3D representation of the structural unit for which the parameters have been determined with the chameleon section (red) highlighted—3BBN‐K. (C) *T* (blue), *O* (red), and *M* (green) distribution for the *K* value (given in the legend) describing the status of protein engaged in metal transport (1CX8). (D) 3D representation of the structural unit for which the parameters have been determined with the chameleon section (red) highlighted—1CX8. (E) *T* (blue) and *O* (red) distribution for the chameleon section in 3BBN‐K. (F) *T* (blue) and *O* (red) distributions for the chameleon section in metal transport protein (1CX8). The blue dots on the *X*‐axis in A and C—chameleon fragment. The 3D structures generated using the VMD program [31, 49].

**Fig. 7:**
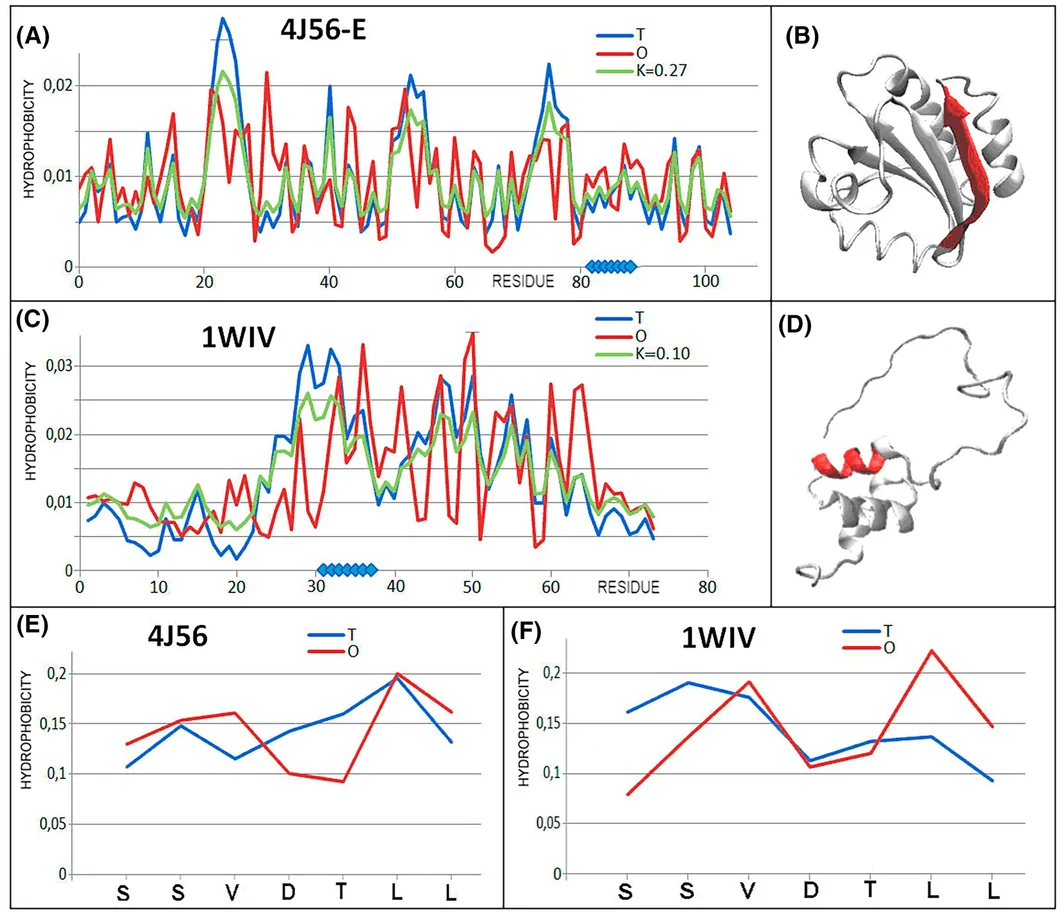
Pair characterisation (Position 5 in Fig. 3C position 280 on the ChSeq database). (A) *T* (blue), *O* (red), and *M* (green) distribution for the *K* value (given in the legend) describing the status of oxidoreductase (4J56). (B) 3D representation of the structural unit for which the parameters have been determined with the chameleon section (red) highlighted – 4J56. (C) *T* (blue), *O* (red), and *M* (green) distribution for the *K* value (given in the legend) describing the status of protein of unknown function (1WIV). (D) 3D representation of the structural unit for which the parameters have been determined with the chameleon section (red) highlighted—1WIV. (E) *T* (blue) and *O* (red) distribution for the chameleon section in 4J56. (F) *T* (blue) and *O* (red) distributions for the chameleon section in protein of unknown activity (1WIV). The blue dots on the *X*‐axis in A and C—chameleon fragment. The 3D structures generated using the VMD program [31, 49].

The localisation of chameleon sections is varied in the pairs discussed.

A special example is a set of two chameleon proteins: ligase—domain (0‐302) (3N05) and ligand binding protein (2RB3). Domains containing chameleon fragments show a status expressed with very low RD values, indicating a high match with the micelle‐like system. The status of chameleon fragments is expressed with extremely low RD values, with even lower RD values for the chameleon fragments. This indicates a very high match with the micelle‐like system. It can be concluded that chameleon sections stabilise the status of the arrangement with a centric hydrophobic core and a polar surface (Fig. 8; example 6).

**Fig. 8:**
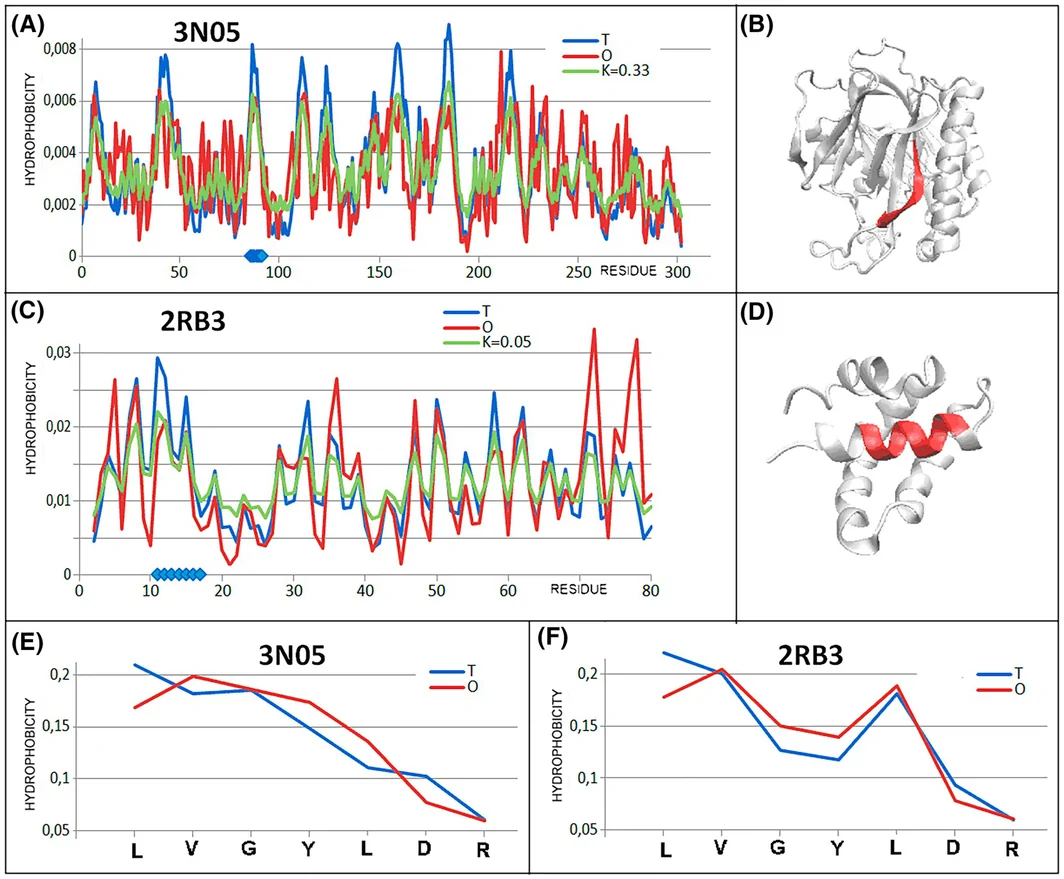
Pair characterisation (example 6 in Fig. 3C position 177 on the ChSeq database). (A) *T* (blue), *O* (red) and *M* (green) distribution for the *K* value (given in the legend) describing the status of ligase (3N05). (B) 3D representation of the structural unit for which the parameters have been determined with the chameleon section (red) highlighted—3N05. (C) *T* (blue), *O* (red), and *M* (green) distribution for the *K* value (given in the legend) describing the status of lipid‐binding protein (2RB3). (D) 3D representation of the structural unit for which the parameters have been determined with the chameleon section (red) highlighted—2RB3. (E) *T* (blue) and *O* (red) distribution for the chameleon section in 3N05. (F) *T* (blue) and *O* (red) distributions for the chameleon section in 2RB3. The blue dots on the *X*‐axis in A and C—chameleon fragment. The 3D structures generated using the VMD program [31, 49].

An opposite example of the role of chameleon sections is the arrangement labelled 1 (Fig. 4) – proteins: isomerase/lyase (PDB ID 1I7O) and a protein representing the immune system (PDB ID 2A73‐B). The chameleon sections included in the micelle‐like status domains show a status with local disorder in the sense of a micelle‐like arrangement. High RD values for chameleon sections suggest a locally high disordered status. An interpretation of this status suggests an encoded intentionality of the structural arrangement of chameleon sections. The location of chameleon sections suggests an expected contribution to the structure of the hydrophobic core (Fig. 4). The local distribution of hydrophobicity in these sections shows a high mismatch with the expected status. It can be speculated that the role of these chameleon sections is to introduce local destabilisation of the hydrophobic core. The profiles (Fig. 4) indicate the opposite arrangement to that expected.

A similar arrangement of protein domains (labelled in Fig. 5 as position 2) with low RD values (micelle‐like arrangement) is present in the proteins: transport protein – Ras‐related protein rab‐2a (PDB ID 1Z0A‐A) and hydrolase (PDB ID 3N1D). The status of the domains suggests the presence of a micelle‐like arrangement (low RD values for the domains) at high RD values determining the status of chameleon sections. This means the introduction of a local mismatch with the micelle‐like arrangement. In the case of 1Z0A, the mismatch involves too low a level of hydrophobicity within the hydrophobic core, where high levels are expected for the core‐building residues. As before, it can be speculated that the role of this section is to weaken the stabilisation resulting from the presence of the hydrophobic core. In contrast, in the case of hydrolase (3N1D), the mismatch involves excessive hydrophobicity at a location where the hydrophobicity level is already expected to decrease. In this case, weakening of the core can also be speculated, this time involving the possibility of a displacement of the chameleon section toward the hydrophobic core.

### Chameleon section with a length of 9 aa with a partially present helical and β‐structured form

The last pair of chameleon proteins discussed here (example 4 – Fig. 9) containing a fragment with the sequence (GKIRDSNRS) and thus with a length of 9 aa is: Gephyrin. Fragment: C‐terminal domain also referred to as putative glycine receptor‐tubulin linker protein (glycine receptor 58 kda subunit) (PDB ID – 1T3E) and membrane protein (PDB ID 1S0P). This pair of proteins is an exception from a viewpoint of secondary structure. The chameleon section has a varied structure containing N‐terminal five residues with a beta‐structure and then a loop is present and the last two residues form a helix arrangement. In contrast, in the membrane protein, the first five residues represent the helical form, while the remaining residue forms a loop.

**Fig. 9:**
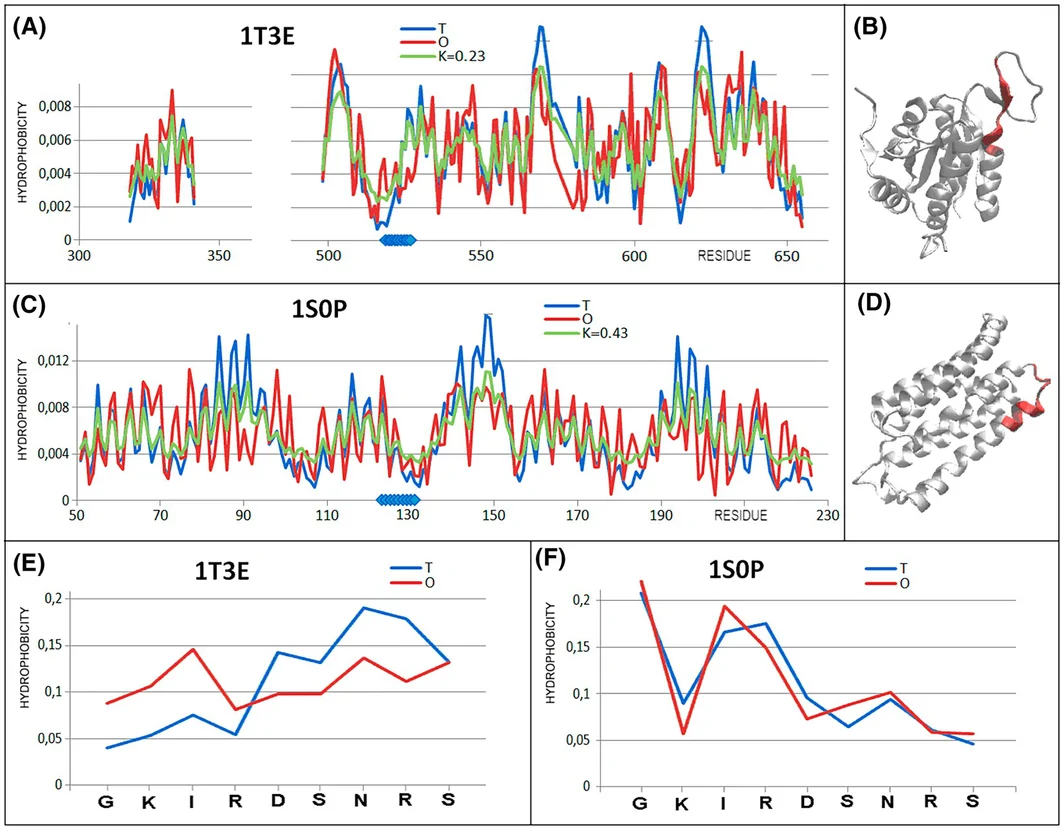
Pair characterisation (4 in Fig. 3C position 3 on the ‘Table of loose criterion’ on ChSeq database). (A) *T* (blue), *O* (red) and *M* (green) distribution for the *K* value (given in the legend) describing the status of glycine receptor (1T3E). (B) 3D representation of the structural unit for which the parameters have been determined with the chameleon section (red) highlighted – 1T3E. (C) *T* (blue), *O* (red), and *M* (green) distribution for the *K* value (given in the legend) describing the status of membrane protein (1S0P). (D) 3D representation of the structural unit for which the parameters have been determined with the chameleon section (red) highlighted – 1S0P. (E) *T* (blue) and *O* (red) distribution for the chameleon section in 1T3E. (F) *T* (blue) and *O* (red) distributions for the chameleon section in 1S0P‐A. The blue dots on the *X*‐axis in A and C—chameleon fragment. The 3D structures generated using the VMD program [31, 49].

Both domains in question show a high micelle‐like ordering, whereas the chameleon sections show a radically different status from the point of view of their contribution to the micelle‐like structure. The status of the chameleon section in the membrane protein shows an extremely high match between the *O* distribution and the *T* distribution. The chameleon section is located in the transition zone between the chain loop exposed to the aqueous environment with the second end section already belonging to the contact zone in the hydrophobic membrane. As shown by the set of *T* and *O* profiles (Fig. 9), the *O*_*i*_ level almost overlaps with that of the corresponding *T*_*i*_ values. Here, the chameleon section participates in the construction of the micelle‐like organisation of hydrophobicity. A completely different status is shown by the chameleon section in the glycine receptor, where *O*_*i*_ levels are opposite to the expected *T*_*i*_ levels (Fig. 9). The role of the chameleon section appears to be involved in the stabilisation of the loose loop.

Figure 10 visualises the status of the chameleon section within the dimer. The chameleon section is responsible for contact with the B‐chain and the exposed loop plays an important role in this stabilisation. Domain (green) with chameleon fragment (green spheres) in chain A (with differentiated domains) in dimer with chain B (white).

**Fig. 10:**
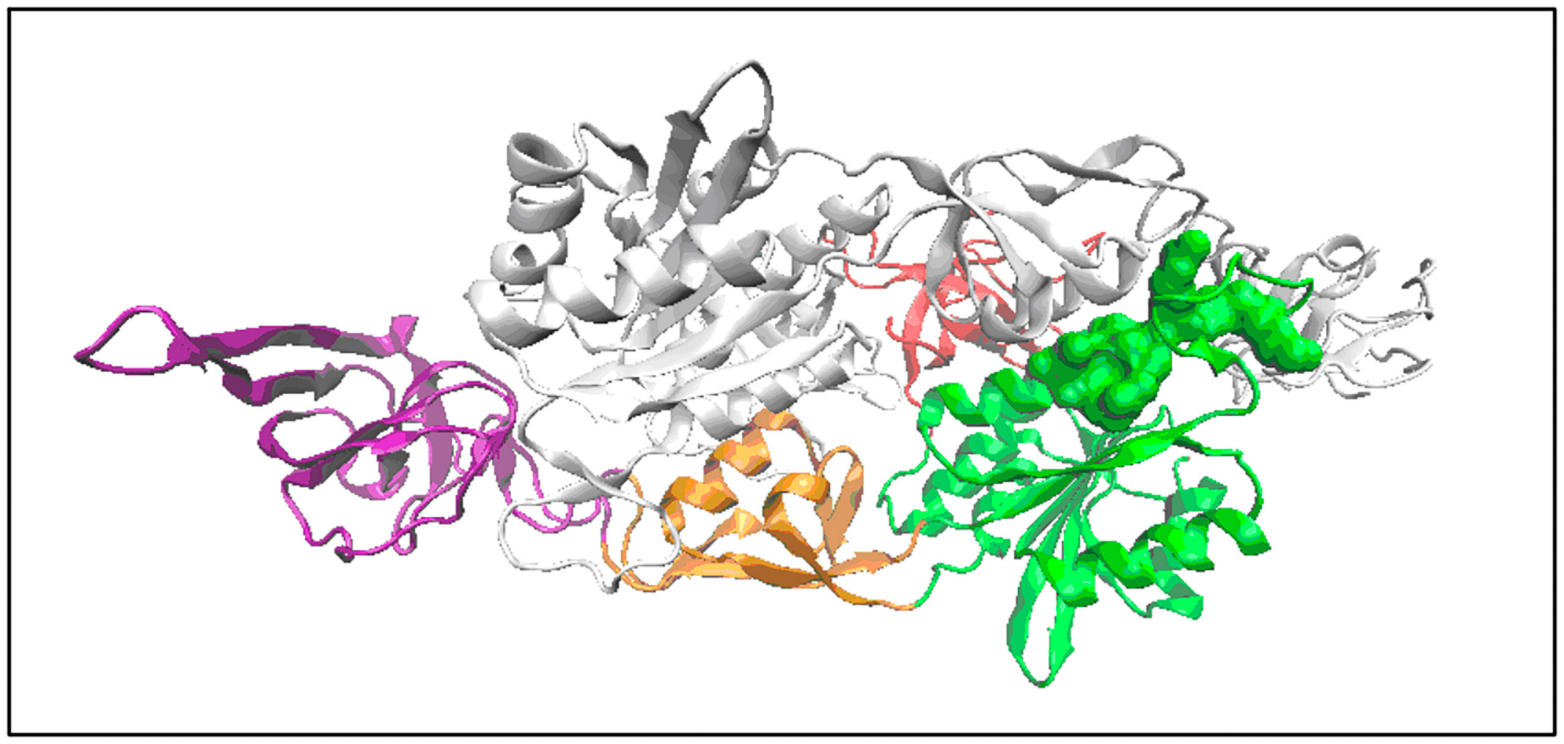
The 3D structure of (1T3E) with domains distinguished by colours. The status of the chameleon section (green spheres) within the green domain. Homo‐dimer (1T3E): chain A with domains distinguished by different colours; chain B—white. The chameleon fragment is part of the domain highlighted as green spheres in the green domain. The involvement of the chameleon section in the interaction with the B‐chain is visible. The 3D structure generated using the VMD program [31, 49].

Examples labelled in Fig. 3C as positions 3, 4 and 5 show common characteristics, where the status of the chameleon section of one protein is different from the status in the other protein. It should be noted that both proteins/domains show a high degree of match with the micelle‐like system. The status of the chameleon sections shows their role as supporting a micelle‐like arrangement (sections with low RD values) with a completely different status of the chameleon section in the other protein in a chameleon protein pair.

An extremely high RD value for the chameleon section is present in the case of 3BBN‐K (example 3). This is a protein that is part of the complex ribosome system. The chameleon section is involved in numerous interactions with other chains of this complex. This section, which is subject to the inter‐chain system, does not locally fulfil the conditions resulting from structuring consistent with a micelle‐like arrangement. The structuring of the chameleon section in the membrane protein (1CX8) turns out to meet expectations, showing a low RD value.

The pair of proteins identified as position 5 in Fig. 7 proteins: 4J56‐E and 1WIV reveals an extremely low RD value for the chameleon section in the protein of unknown status. This protein is an example of near‐perfect ordering according to the micelle‐like model. The status of 4J56‐E is distinguished by a relatively high RD value indicating a local mismatch with a micelle‐like arrangement.

## Discussion

The presence of chameleon sequence is just the effect of evolution. The probability calculation may estimate the probability for 20 units (amino acids) to produce the identical sequence of these units. The analysis of the complete set of proteins as they are available in PDB (246031 Dec 10, 2025) produced the ChSeq data base collecting the proteins with chameleon sequences. The specialists in evolution shall focus their attention on the question of why only a few pairs of proteins representing the length of chameleon sequence containing 6–10 amino acids long fragments are present in the large set of proteins.

The sequence of amino acids as well the 3D structure is generated by evolution to produce the aim‐oriented constructions to ensure the biological activity of the protein. This is why the probability calculus (based on randomness) is not applicable for the analysis of amino acids selection in proteome.

There is no possibility to predict the chameleon sequences using our FOD‐M model. However, our analysis may be used for those specialists who are engaged in the construction of *de novo* designed proteins using the information regarding sequences and their preferences in respect to secondary structure. Our method may help to predict the position of certain chain fragments in the protein body using the construction of a hydrophobic core as the criterion, as it is shown in [24].

When discussing secondary structure relationships in chameleon sections, it is important to include a discussion on the comparative analysis of proteins that differ in point mutations, which results in the conversion of a helical‐form‐dominated structure into a β‐structure‐dominated form [24, 25, 26, 27].

The match of secondary structure has been shown to be not the priority aim for these proteins. Achieving a micelle‐like status with a centrally located hydrophobic core appears to be paramount. The proteins discussed here show very low RD values by adopting alternately a helical and β‐structured form [24, 28, 29, 30]. The other groups of chameleon proteins are discussed in [18, 31]. The role of the hydrophobic core structure was considered in the analysis of the impact of the introduced mutation [32].

The examples of proteins given in this part of the paper demonstrate the superior role of global ordering (in this case highly consistent with a micelle‐like arrangement of hydrophobicity distribution in protein body) against amino acid sequence‐based preferences. The appropriate form of secondary structure is a means to the goal of adapting structuring to an aqueous environment with a centrally located hydrophobic core and a polar surface.

Amyloid transformation introducing a predominant contribution of Beta structures is particularly emphasised in the discussion of the preferred secondary structure. This transformation occurs while keeping the amino acid sequence unchanged. The comparative analysis based solely on the secondary structure in context of amyloid transformation leads to a predisposition analysis for each of the structures discussed [33, 34, 35, 36].

The comparison of the statuses of the respective native and amyloid forms is possible for proteins for which both forms are available: α‐synuclein, the V‐domain of light chain of IgG, and transthyretin. Specifying their status suggests the significant importance of environmental characteristics and its influence on this transformation [37, 38, 39, 40].

The set of these proteins demonstrates the organisation of the entire system subordinating the match of the secondary structure appropriate to the constructed system [41, 42, 43].

The observations presented in this paper suggest the necessary modification in models applied for protein structure prediction. The minimisation of energy expressing the internal interactions to search for optimal protein structure is not sufficient. The important role of environment shall be present in models applied in search for protein structures. The multi‐object optimisation is suggested [44]:

(7) $$F\left(r_{\mathrm{ij}}\right)=F\left(f_{\mathrm{INT}}\left(r_{\mathrm{ij}}\right),f_{\mathrm{EXT}}\left(r_{\mathrm{ij}},K\right)\right)$$

Where *F*(*r*_*ij*_) is function expressing the energetical status of protein dependent on the r_ij_ distances between interacting atoms. The f_INT_(*r*_*ij*_)—the internal force field expressing the non‐bonding interactions (electrostatics, vdW and torsional potential) dependent on *r*_*ij*_—distance between interacting atoms and f_EXT_(*r*_*ij*_, *K*)—external force field dependent on *r*_*ij*_ distances between interacting atoms and dependent on *K* representing the external conditions as it is defined by the *M* function (Eqn 6). Contradiction between INT and EXT force fields makes the minimisation procedure complicated. The decrease of INT function causes the increase of EXT function and vice versa. This is why the special procedure shall be applied: front Pareto [45]. This method allows identification of the consensus between these two functions. The application of such model is planned in a close future to *In Silico* simulation of protein folding process as environment dependent.

Discussion on the detailed energetics of the protein structure is widely presented issue. The search for optimal energetics, which is the standard criterion used in many force fields oriented on protein structure prediction is expressed by E_INT_ in our model. This issue especially focused on chameleon proteins is the object of the paper [46]. We analyse the hydrophobic interactions as factors responsible for structural stabilisation so far omitted in procedures oriented on protein structure prediction.

Analysis of chameleon structures discusses the different secondary forms of chain fragments of the same sequence representing helical and Beta‐structural forms. The status of these fragments appears similar independently of the secondary forms. The conclusions are limited to the similar status of the chameleon fragment independently of the secondary structure. However, the large scale of structural transformation always leading to Beta‐structural forms is observed in amyloid transformation. Independently of the original (native) structural form of the chain, it gets changed to Beta‐structural forms. This transformation requires significant changes in external conditions expressed by *K* values in the FOD‐M model. The population analysis of transthyretin amyloids is presented in [47]. The changes of *K* = 0.5 for transthyretin in native form to the range of *K* = (1.5–1.9) are observed for amyloid structures as they are available in the PDB. These changes are certainly linked with the changes in the external force field. It is hard to speculate what factor of the human body is able to represent such significant environmental conditions. The mutation‐dependent amyloid transformation of transthyretin (aggressive and resistant) forms is discussed in [48].

## Conclusions

The postulate of non‐local targeting vs. broader context‐dependent adoption of the appropriate form of secondary structure, referred to as environment‐dependent, was presented in [39]. This implies that folding is also dependent on other factors, including environmental factors in particular, in which the same sequence can acquire different secondary forms. This work has demonstrated the superior role of the construction of a hydrophobicity distribution system common to the whole protein molecule by reproducing a micelle‐like system targeted by the aqueous environment. The low values of the *K* parameter in the model used suggest a critical role of the polar aqueous environment in the folding of the proteins in question. The adoption of the appropriate form of secondary structure is of lower importance, subject to the micellation mechanism characteristic of bi‐polar molecules, such as amino acids. It is therefore impossible to speak of the predisposition of a given sequence to a given secondary structure without taking into account the influence of the immediate environment on its structuring. Different external conditions (external force field) actively involved in the folding process appear to play a dominant role in relation to the predisposition of the amino acid sequence itself. This supports the statement that any chain can convert to amyloid under specific conditions [42, 43].

In relation to the model presented here, it takes a quantitative form as an external force field‐dependent transformation, which is seen in the examples of chameleon sequence proteins.

## Conflict of interest

The authors declare no conflict of interest.

## Author contributions

Conceptualization: IR, LK; Calculations: KS; Data curation: KG, PN. All Authors read and approve the final version of the manuscript.

## Data Availability

The program allowing recalculation of all presented results is available in open access system. The addresses are given in Materials and Methods.
